# Rational Design Strategies for Stimuli‐Responsive DNAzymes Using Modified and Artificial Nucleotides

**DOI:** 10.1002/chem.202503570

**Published:** 2026-01-21

**Authors:** Yusuke Takezawa, Mitsuhiko Shionoya

**Affiliations:** ^1^ Chemistry and Materials Program College of Engineering, Shibaura Institute of Technology Saitama Japan; ^2^ Research Institute for Science and Technology Tokyo University of Science Noda‐shi Chiba Japan

**Keywords:** DNA, DNAzyme, modified nucleobase, oligonucleotides, switching

## Abstract

DNA, traditionally regarded as genetic material, has emerged as a versatile building block in molecular technology. Catalytic DNA oligomers known as DNAzymes, especially those capable of cleaving target RNA at specific sites, have shown great potential in DNA‐based diagnostics, therapeutics, and dynamic DNA nanotechnology. For advanced applications, stimuli‐responsive DNAzymes, which are activated only under specific conditions or by specific chemical stimuli, have attracted particular attention. Although such DNAzymes can be obtained by in vitro selection under strictly controlled conditions, more general strategies for their rational design are strongly needed. In this approach, stimuli‐responsiveness is introduced into existing DNAzymes by sequence engineering or chemical modification. This review focuses on the rational design of stimuli‐responsive RNA‐cleaving DNAzymes using modified and artificial nucleotides. Here, we discuss representative strategies, including recent examples: (i) stimulus‐induced reconstitution of split DNAzymes, (ii) activity control by strand cleavage or ligation, (iii) blocking of the catalytic core with removable oligonucleotides, (iv) caging with labile protecting groups, and (v) stimuli‐induced conformational switching between inactive and active structures. These approaches enable externally controllable activation mechanisms, while maintaining the intrinsic catalytic activity of DNAzymes, providing a valuable toolkit for molecular sensing and DNA nanotechnology.

## Graphical abstract

Activity control of RNA‐cleaving DNAzymes in response to external stimuli has attracted growing interest for applications in DNA‐based diagnostics, therapeutics, and dynamic DNA nanotechnology. This review summarizes rational design strategies for stimuli‐responsive DNAzymes using modified or artificial nucleotides, including split and caged DNAzymes, as well as systems that undergo stimuli‐induced structural transformations.

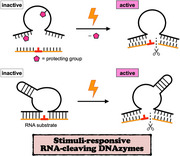

**Abbreviations**
1MeA: *N* ^1^‐methyladenine
3MeC: *N* ^3^‐methylcytosine
AD: adamantane
BO: phenylboronate
caU: 5‐carboxyuracil
CB[7]: cucurbit[7]uril
^CNV^K: 3‐cyanovinylcarbazole‐type artificial nucleobase
DEACM: 7‐diethylaminocoumarin
DNAzyme: deoxyribozyme
εA: 1,*N* ^6^‐ethenoadenine
EDTA: ethylenediaminetetraacetic acid
FTO: fat mass and obesity‐associated protein
GHK: Glycyl‐histidyl‐lysine (a Cu^II^‐binding peptide)
G^NB^
: *O* ^6^‐nitrobenzyl guanine
H: hydroxypyridone‐type artificial nucleobase
HEPES: 4‐(2‐hydroxyethyl)‐1‐piperazineethanesulfonic acid
Im^C^
: 4‐carboxyimidazole‐type artificial nucleobase
m^6^A: *N* ^6^‐methyladenine
NBOP: 2‐(2‐nitrobenzyl)oxyphenyl
MGMT: *O* ^6^‐methylguanine DNA methyltransferase
NPOM: 6‐nitropiperonyloxymethyl
*O* ^6^MeG: *O* ^6^‐methylguanine
rA: adenosine ribonucleotide
ROS: reactive oxygen species
SELEX: systematic evolution of ligands by exponential enrichment
TEEP‐OH: thioether‐enol phosphate, phenol substituted (photoremovable thioether‐enol phosphotriester group)
*T*_m_
: melting temperature
PS: photocleavable spacer or phosphorothioate
U^OH^
: 5‐hydroxyuracil

## 1 Introduction

DNA is a biopolymer that stores genetic information, but in recent years, there has been growing interest in applications beyond its original role. DNA‐based molecular technologies have made great advances, including the construction of nanoarchitectures using sequence‐specific hybridization and the development of molecular computing circuits based on strand displacement reactions [1, 2, 3, 4]. Furthermore, the discovery of functional nucleic acids such as DNA aptamers, which selectively bind to small molecules, and DNAzymes, which exhibit catalytic activity, has further expanded the potential of nucleic acid technology [5].

**FIGURE 1:**
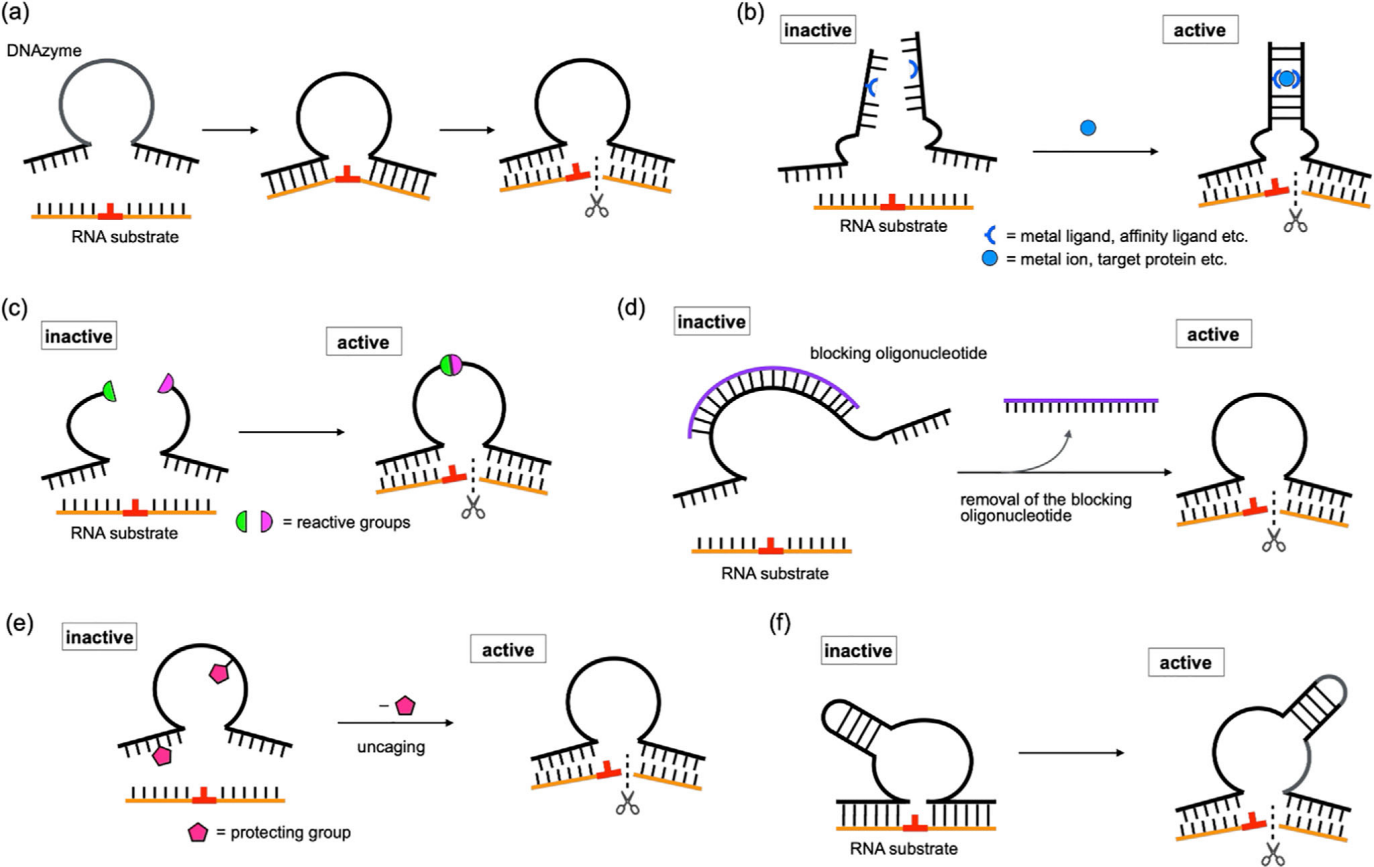
Molecular designs of stimuli‐responsive DNAzymes. (a) Schematic diagram of a native RNA‐cleaving DNAzyme. The cleavage site of the RNA substrate is shown in red. (b) Split DNAzyme whose activity is restored by reassembly of the fragments in response to a stimulus. (c) Stimuli‐responsive DNAzyme activated through ligation of its backbone. (d) Stimuli‐responsive DNAzyme in which the catalytic core is blocked with a removable complementary oligonucleotide. (e) Caged DNAzyme whose activity is recovered upon removal of photolabile or chemically labile protecting groups. (f) Stimuli‐responsive DNAzyme activated through intramolecular structural transformation.

DNAzymes are short, single‐stranded DNA molecules capable of catalyzing chemical reactions and can be considered the DNA counterpart of naturally occurring ribozymes [6, 7]. Since the first DNAzyme was reported by Joyce in 1994 [8], a wide variety of DNAzymes have been developed using Systematic Evolution of Ligands by EXponential enrichment (SELEX), an in vitro selection technique used to isolate functional nucleic acids with desired properties. Among these, RNA‐cleaving DNAzymes, which bind to target RNA sequences and catalyze site‐specific cleavage, have been particularly widely studied (Figure 1a) [9]. A typical RNA‐cleaving DNAzymes consists of two substrate‐binding arms with base sequences complementary to the target RNAs and a folded catalytic core containing several nucleobases that function as acid–base catalysts, similar to protein enzymes. Most of these DNAzymes require metal ion cofactors to promote cleavage of phosphodiester bonds. RNA‐cleaving DNAzymes have shown great promise in diagnostic and therapeutic applications [10, 11, 12] and dynamic DNA nanotechnology [6, 13, 14].

Toward more advanced and practical applications, there has been growing interest in developing DNAzymes that exhibit catalytic activity only under specific chemical environments or in response to specific external stimuli [15]. Such stimuli‐responsive DNAzymes are highly desirable for a wide range of applications, including biosensing, intracellular analysis, and the construction of dynamic DNA‐based molecular devices. Although it is possible to isolate DNAzymes that respond to specific stimuli using SELEX under tightly controlled conditions, this approach lacks versatility and requires significant experimental effort for each new target stimulus. A more general and versatile strategy is rational design, whereby stimuli‐responsiveness is introduced into previously identified DNAzyme sequences by deliberate sequence engineering or chemical modification [16, 17]. To date, DNAzymes that respond to a variety of stimuli, including light, metal ions, redox states, and other molecular signals, have been rationally designed based on this approach (see below).

This review surveys rational design strategies for conferring stimuli‐responsiveness to DNAzymes by incorporating chemically modified nucleobases and nonnatural structural motifs. These strategies expand the functional repertoire of DNAzymes beyond what can be achieved with natural nucleic acid components alone. Here, we present five representative examples of stimuli‐responsive DNAzyme designs, focusing on RNA‐cleaving DNAzymes, one of the most widely studied and practically useful classes of catalytic nucleic acids (Table 1) [9]: (i) splitting the DNAzyme into inactive fragments that reconstitute upon external stimuli (Figure 1b), (ii) controlling activity by strand cleavage or ligation (Figure 1c), (iii) blocking the catalytic core with removable or replaceable complementary oligonucleotides (Figure 1d), (iv) caging the DNAzyme with photolabile or chemically labile protecting groups (Figure 1e), (v) inducing conformational switching between inactive and active structures in response to external signals (Figure 1f). These approaches enable DNAzymes to acquire externally controllable activation mechanisms while maintaining their intrinsic catalytic activity. Such designs hold great promise for a wide range of applications in DNA‐based research and technological innovation.

**TABLE 1:** Design strategies of stimuli‐responsive DNAzymes.

Design strategy	Modification	Stimulus	Key driving force	Characteristics	References
Reassembly of Split DNAzymes	Additional DNA sequence	Oligonucleotides	Nucleic acid hybridization	Ease of preparation; intracellular application	[19, 20]
	DNA aptamer	Small molecule	Aptamer–ligand binding	Ease of preparation	[21]
	Affinity ligand	Target protein	Ligand–protein interaction		[22]
	Cytosine (C)‐rich sequence	H^+^ (pH changes)	Formation of i‐motif structure	Ease of preparation	[23]
	Metal‐ligand‐type nucleobase	Metal ion	Metal‐mediated base pairing	Reversible	[30, 33, 36, 38]
Activation via strand break or ligation	Photocleavable linker	UV irradiation	Photolysis	Irreversible	[42]
	Boronic acid and diol	pH change; catechol	Ester formation and hydrolysis		[44]
Competitive binding of blocker oligonucleotides	Additional DNA sequence	oligonucleotides	Strand displacement	Ease of preparation; application to logic‐gate operations	[45, 46, 47]
	Photocleavable linker	UV irradiation	Photolysis	Irreversible	[42]
	Phosphorothioate linkage	HClO	Oxidative cleavage	Ease of preparation; intracellular application	[48]
	3‐Cyanovinylcarbazole (^CNV^K) nucleotides	UV irradiation	Reversible covalent crosslinking	Reversible; ultrafast response	[50]
	Photocaged nucleobases	UV irradiation	Photo‐deprotection (uncaging)	Irreversible	[54]
	Azobenzene derivatives	UV or visible light irradiation	*Cis–trans* isomerization	Reversible; application to protein expression control	[55]
Caging strategies	Photoremovable protecting group (on the 2’‐OH of the substrate)	UV irradiation	Photo‐deprotection	Irreversible; intracellular application	[39, 61, 63, 64, 65]
	Photoremovable protecting groups (on nucleobases)	UV irradiation	Photo‐deprotection	Irreversible	[53, 66]
	Phenyl boronate‐protected phosphorothioate linkage	H_2_O_2_	Oxidative deprotection	Irreversible; intracellular application	[48]
	*O* ^6^‐nitrobenzyl guanine (G^NB^)	Na_2_S_2_O_4_	Reductive deprotection	Irreversible	[68]
	Phosphorothioate linkage protected with a photoremovable group	UV irradiation	Photo‐deprotection	Prepared via post‐synthetic modification; intracellular application	[70, 71]
	4‐Azidobenzyl group	H_2_S	Reductive deprotection	Prepared via post‐synthetic modification; intracellular application	[72, 73]
	Methylated nucleobases	Enzyme (demethylase)	Enzymatic demethylation	Naturally occurring modifications	[74, 77]
	Guest molecule (e.g., adamantane)	Host molecule (e.g., cucurbit[7]uril); competing guest molecule	Host–guest interaction	Reversible	[78]
Intramolecular structural switching	Azobenzene derivatives	UV or visible light irradiation	*Cis–trans* isomerization	Reversible	[81, 82, 83]
	Metal ligand‐type nucleobase	Metal ion	Metal‐mediated base pairing	Reversible; application to metal ion sensing and logic‐gate operations	[84, 88, 89, 91]
	5‐Modified uracil (U^OH^ and caU)	Metal ion	Switching between hydrogen‐bonded and metal‐mediated base pairs	Reversible	[97, 99]

## 2 Stimuli‐Responsive Split DNAzymes

**FIGURE 2:**
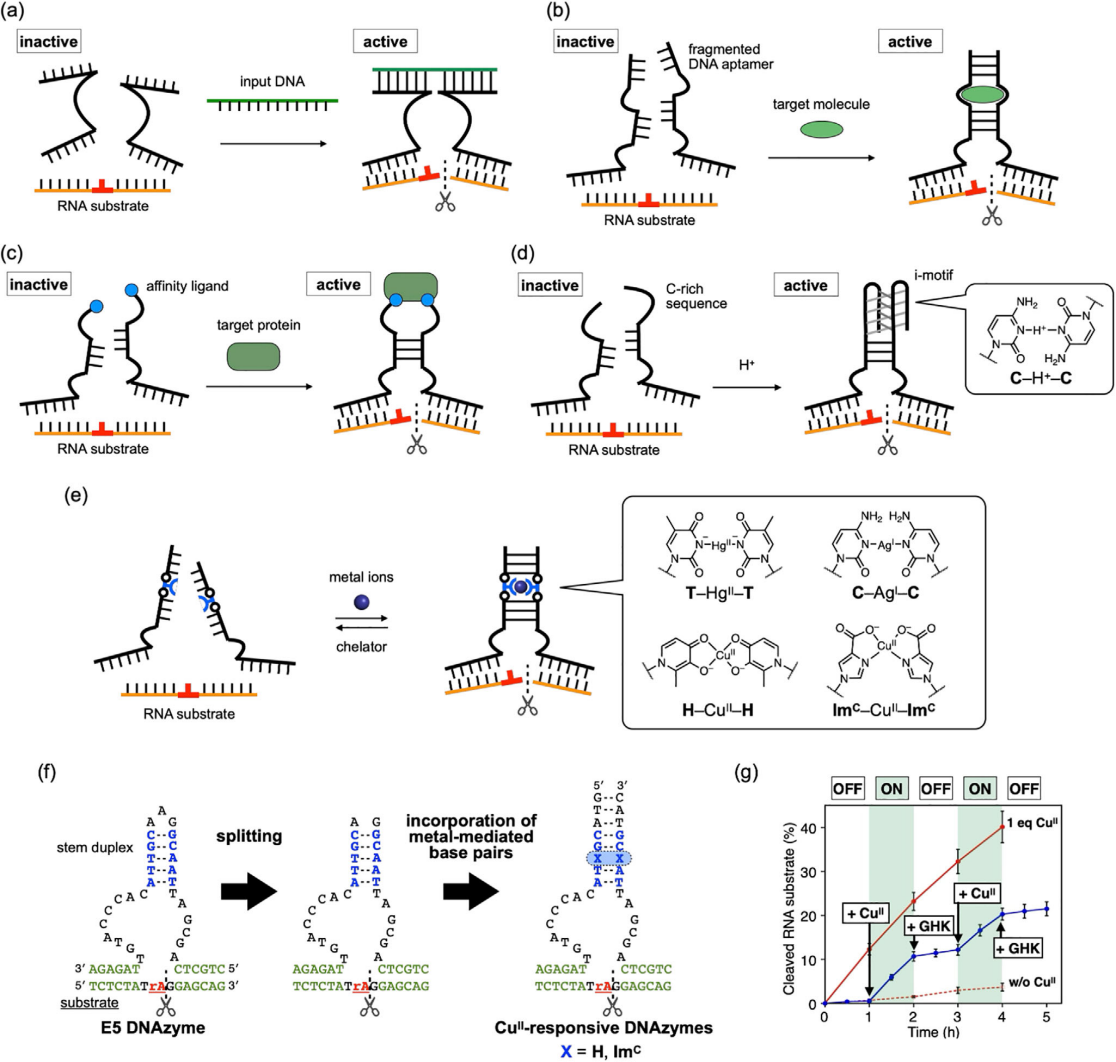
Molecular design of stimuli‐responsive split DNAzymes. (a) Schematic diagram of a split DNAzyme activated by the addition of a target oligonucleotide. The cleavage site of the RNA substrate is shown in red. (b) Schematic diagram of a split DNAzyme tethered to a fragmented DNA aptamer, which is activated in the presence of target molecules. (c) Schematic diagram of a split DNAzyme modified with affinity ligands, which is activated in the presence of target proteins. (d) Schematic diagram of a split DNAzyme tethered to a C‐rich sequence, which is activated under acidic conditions through the formation of an i‐motif structure. (e) Schematic diagram of a metal‐responsive DNAzyme containing metal‐mediated artificial base pairs. (f) Design of Cu^II^‐responsive split DNAzymes based on H–Cu^II^–H and Im^C^–Cu^II^–Im^C^ base pairing. (g) Iterative switching of the activity of an Im^C^‐modified split DNAzyme. Alternate addition of Cu^II^ (1 equiv relative to the DNAzyme), GHK (2 equiv), Cu^II^ (2 equiv), and GHK (4 equiv) was performed. [DNAzyme] = 1.0 µM, [substrate] = 10 µM (10 equiv) in 10 mM HEPES buffer (pH 7.0), 1 M NaCl, 10 mM MgCl_2_, 25 °C. *N* = 3. The DNAzyme activity in the presence (red solid line) and absence of Cu^II^ ions (red dotted line) are also shown. Error bars indicate standard errors. Reproduced with permission from ref. [36]. Copyright 2020 Wiley‐VCH.

One of the simplest strategies for designing stimuli‐responsive DNAzymes is to split and modify a known DNAzyme sequence so that the segmented subunits assemble into its original active structure only in the presence of a specific stimulus or target molecule [18]. This design concept was first demonstrated in split DNAzymes that are activated by the addition of target oligonucleotides [19, 20]. For example, a parent DNAzyme is split into two fragments, each tethered to a DNA oligomer complementary to one half of a target nucleic acid (Figure 2a) [20]. Since each fragment contains only a portion of the catalytic core, the original DNAzyme activity is lost. The two fragments reassemble upon binding to the target DNA (or RNA), reconstructing the complete DNAzyme structure. As a result, the split DNAzyme is activated only in the presence of the target oligonucleotide. Because target specificity can be easily engineered by altering the base sequence of the binding region, this strategy can be applied to the development of biosensors for detecting various nucleic acids including intracellular RNAs.

Using a similar method, split DNAzymes tethered to fragmented DNA aptamers have been designed (Figure 2b) [21]. In the presence of a target molecule, such as adenosine, the intact aptamer structure is restored upon binding to the target molecule, allowing the two subunits to assemble. As a result, the DNAzyme can be activated by the target molecule recognized by the conjugated aptamer. Incorporating affinity ligands such as biotin into the binding arms also enables split DNAzymes to respond to target proteins (Figure 2c) [22]. Furthermore, by introducing C‐rich sequences into the both split fragments, a pH‐responsive DNAzyme was developed (Figure 2d) [23] Under acidic conditions, the formation of the i‐motif quadruplex containing C–H^+^–C base pairs induces the reorganization of the catalytic core and activates the DNAzyme in a pH‐dependent manner. These examples demonstrate the flexibility and versatility of the split DNAzyme design concept.

Split DNAzymes activated with specific metal ions can be rationally designed by incorporating metal‐mediated unnatural base pairs (Figure 2e) [24, 25]. Metal‐mediated base pairs are formed by interstrand metal complexation of either canonical, modified, or synthetic nucleobases at the opposite positions within a DNA duplex [26, 27, 28]. Since the metal‐mediated base pairing generally enhances the duplex stability [29], it can be exploited to the development of metal‐responsive split DNAzymes.

Willner et al. developed Hg^II^‐ and Ag^I^‐responsive split DNAzymes using metal‐mediated base pairs composed of natural nucleobases (Figure 2e) [30]. Natural thymine (T) and cytosine (C) bases are known to form Hg^II^‐ and Ag^I^‐mediated base pairs (T–Hg^II^–T and C–Ag^I^–C, respectively) through the coordination of their nitrogen atom at the 3‐position [31]. For example, an Hg^II^‐responsive DNAzyme was constructed by splitting the known RNA‐cleaving E5 DNAzyme [32] into two subunits at the loop region not involved in the catalytic function. T–T mismatches were introduced into the stem duplex region as binding sites for the target Hg^II^ ions. The presence of the mismatches inhibits the two split strands from hybridizing with each other. In the presence of Hg^II^ ions, the fragments were reassembled, restoring the catalytically active structure through the formation of T–Hg^II^–T base pairs. The experimental results showed that the DNAzyme activity increased with increasing amounts of Hg^II^ ions. Furthermore, removal of Hg^II^ ions inactivated the DNNAzyme. Similarly, by incorporating C–Ag^I^–C base pairs, an Ag^I^‐responsive split DNAzyme was developed, whose RNA‐cleaving activity was reversibly controlled by the addition and removal of Ag^I^ ions.

Split DNAzymes that respond to other metal ions can also be rationally designed by incorporating synthetic ligand‐type nucleobases that form metal‐mediated artificial base pairs (Figure 2e). Takezawa and Shionoya et al. reported the first example of Cu^II^‐responsive split DNAzyme [33], which contains a Cu^II^‐mediated hydroxypyridone base pair (H–Cu^II^–H). The H–Cu^II^–H base pair is one of the most investigated metal‐mediated base pairs, and its formation has been shown to significantly stabilize DNA duplexes (Δ*T*_m_ = +13 °C) [34]. Specifically, the RNA‐cleaving E5 DNAzyme [32] was split into two segments, and an H–H mismatch pair was introduced into its variable duplex region (Figure 2f). The sequence design was optimized by varying the position of the H–H mismatch and the length of the stem duplex. The result showed that introducing an H–Cu^II^–H pair near the catalytic core reduced the DNAzyme activity due to structural distortion caused by unnatural base pairing. When an H–H mismatch was introduced at the duplex ends, the stem duplex remained stable even without Cu^II^ ions, so DNAzyme activity was unaffected by adding Cu^II^ ions. The most effective response to Cu^II^ ions was observed for a split DNAzyme with an 8‐base‐pair stem in which the H–H pair was located at the third position from the catalytic core. In the absence of Cu^II^ ions, the optimized split DNAzyme exhibited significantly reduced activity. Upon the addition of equimolar Cu^II^ ions, the two split fragments associated via the formation of an H–Cu^II^–H base pair, reconstituting the catalytically active DNAzyme structure. The apparent catalytic activity was found to increase approximately 5.5‐fold. It was also confirmed that one equivalent of Cu^II^ ions is sufficient to activate the H‐modified split DNAzyme. The Cu^II^ ions were removed by adding a Cu^II^‐binding peptide GHK [35], which inactivated the DNAzyme. As a result, the activity of the DNAzyme activity was demonstrated to be repeatedly switched by the alternate addition of Cu^II^ ions and GHK. It should be noted that the activity of the Cu^II^‐responsive DNAzyme was also controlled based on the redox of the copper ions.

Related to the above, a Cu^II^‐responsive split DNAzyme with improved switching efficiency was developed [36]. It is based on the same sequence design but incorporates a different Cu^II^‐mediated base pair, Im^C^–Cu^II^–Im^C^ (Figure 2e, f) [37]. Under the neutral conditions favorable for the DNAzyme reactions, the formation of an Im^C^–Cu^II^–Im^C^ base pair was shown to stabilize DNA duplexes more efficiently (Δ*T*_m_ = +35 °C) than the H–Cu^II^–H base pairing. The catalytic activity of the split DNAzyme containing Im^C^ nucleotides in place of H was significantly suppressed under Cu^II^‐free conditions. This is because the electrostatic repulsion between the negatively charged Im^C^ bases strongly prevents the hybridization of the split subunits. The addition of equimolar Cu^II^ ions reconstituted the split DNAzyme through the Im^C^–Cu^II^–Im^C^ base pair formation, resulting in 12‐fold increase in catalytic activity. Furthermore, the activity of the Im^C^‐modified DNAzyme was reversibly controlled by the addition, removal, and reduction of Cu^II^ ions (Figure 2g).

Another type of DNAzyme that cleaves RNA has been redesigned into a Cu^II^‐responsive split DNAzyme by utilizing one of the damaged nucleobases as metal‐binding sites [38]. Specifically, a DNAzyme known as NaA43 [39] was fragmented and three pairs of 1,*N* ^6^‐ethenoadenine (εA) nucleotides were introduced into its stem duplex. The εA base is an etheno‐adduct of the natural adenine (A) base and has been reported to form a Cu^II^‐mediated εA–Cu^II^–εA base pair in DNA duplexes [40]. It was demonstrated that the activity of the εA‐modified split DNAzyme increased 5.3‐fold with the addition of three equivalents of Cu^II^ ions (i.e., one equivalent for the εA–εA mismatch).

The split DNAzyme design concept has proven useful for conferring responsiveness to various external stimuli. In addition to the examples mentioned above, another strategy for designing split DNAzymes is to segment the substrate‐binding domain and reconstitute it in a stimulus‐dependent manner [19, 41]. This approach restores the substrate recognition ability of the DNAzymes, enabling it to cleave the RNA substrate. As a fundamental and versatile approach, split DNAzymes are expected to play a central role in the future development of stimuli‐responsive DNAzymes.

## 3 Activity Control Through Strand Break or Ligation

**FIGURE 3:**
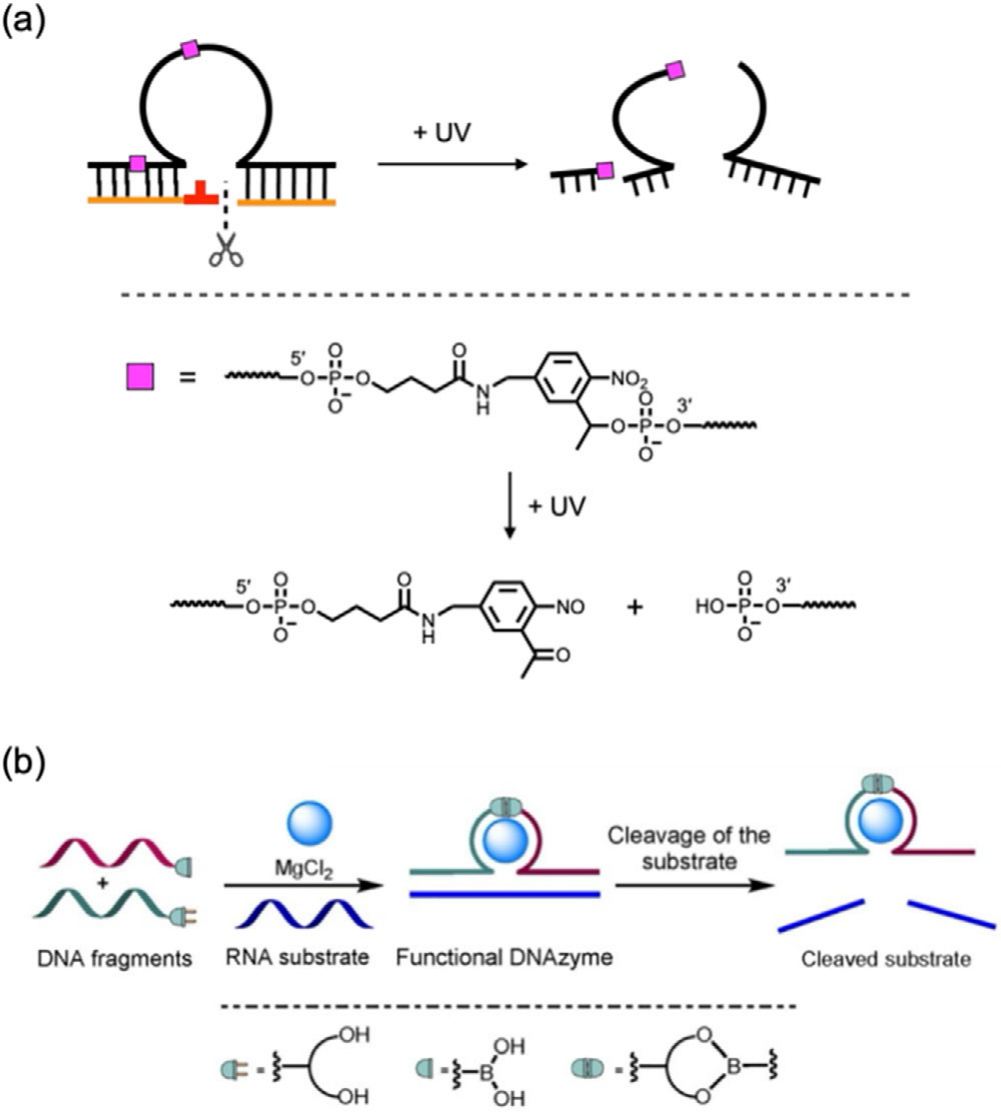
Stimulus‐dependent regulation of DNAzyme activity through strand break or ligation. (a) Photoresponsive DNAzyme containing a photocleavable spacer (PS, shown as a pink square) in the backbone. The structure of the PS moiety and photolysis products are also shown. (b) DNAzyme activity control based on the boronic ester formation within the backbone. Reproduced with permission from ref. [44]. Copyright 2021 Wiley‐VCH.

The activity of DNAzymes can be controlled through stimulus‐dependent cleavage or ligation of the DNAzyme backbone. Dmochowski et al. strategically designed photoresponsive DNAzymes by incorporating a photocleavable spacer (PS) into the DNA backbone (Figure 3a) [42]. The 10–23 DNAzyme [43] was modified by substituting one nucleotide in the catalytic core and one in the substrate‐binding domain with PS. The modified DNAzyme showed a reduced RNA‐cleaving activity compared to the original DNAzyme, but was still active. After UV irradiation, the DNAzyme strand was split into three segments by photolysis of the PS linkage, resulting in complete loss of DNAzyme activity. As a result, the DNAzyme was successfully inactivated by photoirradiation.

Other types of internucleotide linkages have the potential to regulate DNAzyme activity in response to specific stimuli. For example, Smietana et al. introduced a boronate ester into the backbone of the 10–23 DNAzyme (Figure 3b) [44]. The parent DNAzyme was split into two fragments, ideally losing its original activity. One fragment was modified with a boronic acid group at the 5’ end, while the other fragment was modified with a ribonucleotide at the 3’ end, providing a *cis*‐diol group. The formation of a boronate ester was expected to reconstitute a catalytically active full‐length DNAzyme. Although the results were highly dependent on the position of the boronate ester and the type of the boronic acid modification, DNAzyme activation via boronate ester formation was observed in some cases, as expected. It is also noteworthy that the DNAzyme containing a boronate linkage exhibited pH‐dependent activity. Furthermore, its catalytic activity decreased upon addition of an excess amount of catechol, which acts as a competing diol to break the DNAzyme backbone. This example demonstrates the potential utility of artificial backbone linkages in the development of stimuli‐responsive DNAzymes.

## 4 Stimuli‐Responsive DNAzymes With Blocker Oligonucleotides

The action mechanisms of the stimuli‐responsive DNAzymes described above relies on DNA hybridization in response to a stimulus. Alternatively, DNAzyme activity can be controlled by stimuli‐dependent dissociation of DNA duplexes. In this approach, the intrinsic catalytic function of a DNAzyme is abolished by blocking either the catalytic loop or the substrate‐binding domain with a complementary oligonucleotide. The inactivated DNAzyme is expected to regain its original activity when the blocking oligonucleotide is removed by a specific stimulus.

**FIGURE 4:**
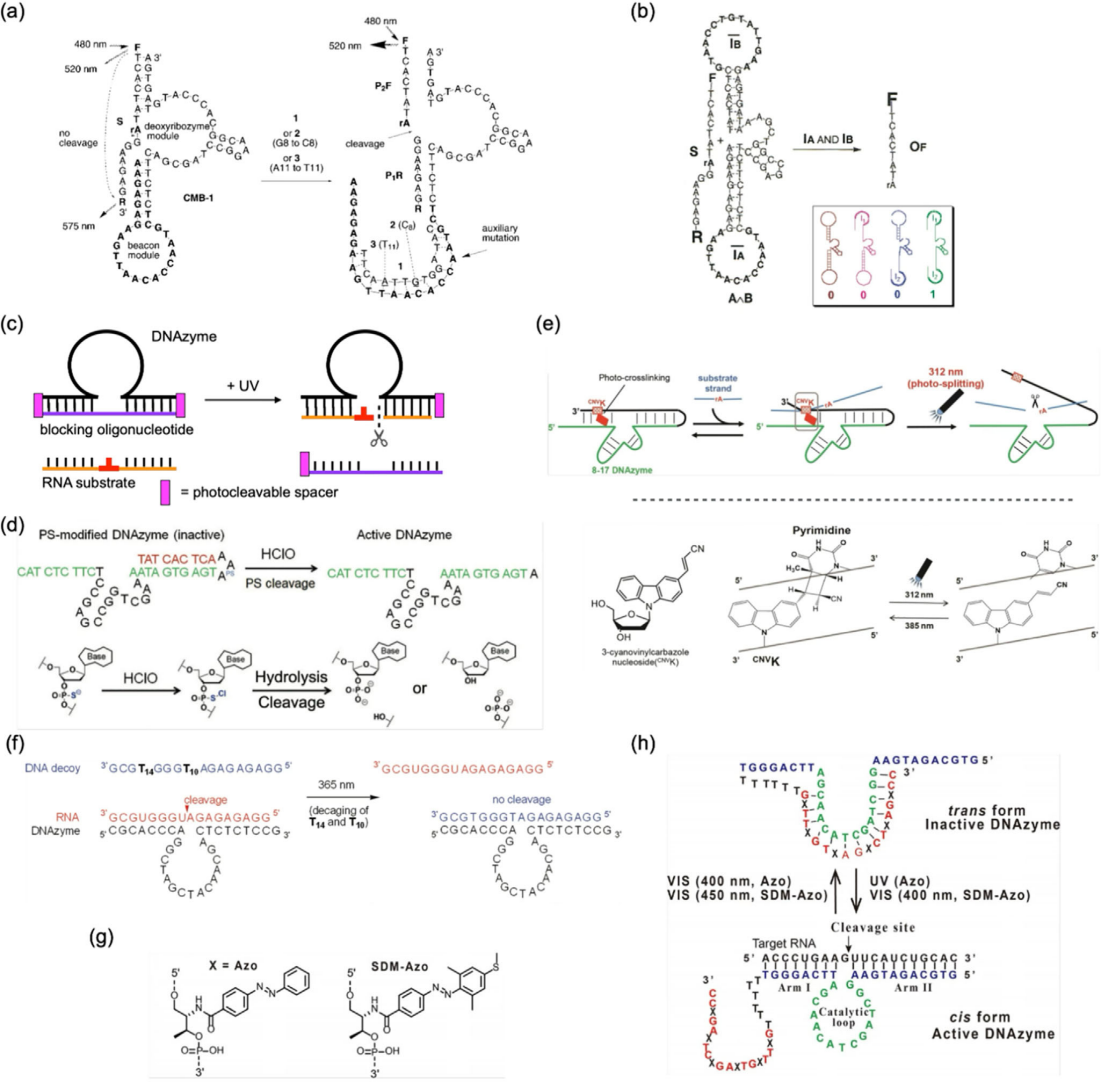
Molecular design of stimuli‐responsive DNAzymes using blocker oligonucleotides. (a) A DNAzyme that is activated by the addition of a specific input oligonucleotide. Binding of input DNA **1** to the beacon module unblocks the substrate‐binding arm and activates the DNAzyme. Reproduced with permission from ref. [45]. Copyright 2001 Wiley‐VCH. (b) An AND‐gate DNAzyme activated by the addition of two input oligonucleotides (I_A_ and I_B_), Reproduced with permission from ref. [46]. Copyright 2002 American Chemical Society. (c) Photoresponsive DNAzyme cyclized via photocleavable spacers (PS, shown as a pink square). See Figure 3a for the chemical structure of PS. (d) A DNAzyme linked to a blocking sequence (red) via a phosphorothioate (PS) linkage (blue). It is activated in the presence of HClO. Reproduced with permission from ref. [48]. Copyright 2019 Wiley‐VCH. (e) A photoresponsive DNAzyme containing a 3‐cyanovinylcarbazole (^CNV^K) nucleobase, which can form covalent crosslinkage with a pyrimidine base on the complementary strand. The chemical structure of ^CNV^K and its photoreversible reaction with a pyrimidine base are also shown. Reproduced with permission from ref. [50]. Copyright 2020 Wiley‐VCH. (f) Photochemical inactivation of a DNAzyme using a caged blocker strand (DNA decoy). T10 and T14 are protected with a photoremovable 6‐nitropiperonyloxymethyl (NPOM) group. See Figure 5b for the chemical structure of NPOM. Reproduced with permission from ref. [54]. Copyright 2010 American Chemical Society. (g) Chemical structures of azobenzene (Azo) and 2,6‐dimethyl‐4‐(methylthio)azobenzene units (SDM‐Azo), which undergo *cis–trans* isomerization upon photoirradiation. (h) Photoresponsive DNAzymes immobilized with a blocker DNA containing Azo or SDM‐Azo units (denoted by X). Figures (g) and (h) are reproduced with permission from ref. [55]. Copyright 2018 Wiley‐VCH.

DNAzymes activated by specific DNA strands can be designed by attaching a stem–loop DNA motif to the DNAzyme (Figure 4a) [45]. In this design, the DNAzyme strand is extended to include a loop domain and an oligonucleotide complementary to one of the substrate‐binding arms, which inhibits substrate binding and suppresses DNAzyme activity. The loop sequence is designed so that hybridization with input DNA opens the stem–loop, in a manner similar to molecular beacons, thereby restoring substrate binding and activating the DNAzyme in response to target DNA. This strategy also has been applied to the development of DNAzymes that demonstrate logic gate operation in response to multiple DNA inputs [46, 47]. For example, an AND‐gate DNAzyme that responds to two input strands can be constructed by introducing stem–loop structures at both termini, each of which blocks the substrate‐binding domain (Figure 4b) [46]. Only when both stem–loops are opened by their respective input DNAs, the DNAzyme can bind and cleave the substrate.

Based on this design principle, Dmochowski et al. developed a photoactivatable DNAzyme [42]. The 10–23 DNAzyme [43] was covalently linked to a blocking strand via photocleavable linkers (Figure 4c). A DNA strand identical to part of the RNA substrate was used as a blocking strand and tethered to both the 5’ and 3’ ends of the DNAzyme. The resulting circular structure completely inactivated the DNAzyme. Cleavage of the linkers by UV irradiation unlocked the substrate‐binding domain and restored the activity of the DNAzyme, demonstrating successful photoinduced activation.

Phosphorothioate (PS) linkages have been used to develop DNAzymes that can be activated by reactive oxygen species (ROS). Xing et al. designed a modified 8–17 DNAzyme [43] in which a blocking strand complementary to one of the substrate‐binding arms was tethered via a PS‐containing linker (Figure 4d) [48]. Because PS bonds are cleaved by hypochlorous acid (HClO) [49], it was expected that treatment with HClO would release the blocker. Addition of 0.5 µM HClO increased RNA‐cleaving activity 12‐fold, whereas other ROS (e.g., H_2_O_2_) did not activate the DNAzyme. This HClO‐responsive DNAzyme was shown to function in living human and mouse cells.

Oligonucleotides that form reversible crosslinks with complementary strands have also been employed to develop stimuli‐responsive DNAzymes. Fujimoto et al. used 3‐cyanovinylcarbazole (^CNV^K) nucleotides [50], which covalently crosslink with pyrimidine bases on complementary strands via a [2+2] photocycloaddition reaction (Figure 4e) [51]. Because this photo‐crosslinking process is ultrafast and reversible, ^CNV^K is well suited for regulating DNA hybridization in response to light [52]. The RNA‐cleaving 8–17 DNAzyme [43] was conjugated with a blocker DNA complementary to the substrate‐binding arms, and a ^CNV^K residue was incorporated into the blocker as a photoresponsive unit. Upon irradiation with UV light at 385 nm, ^CNV^K forms a covalent crosslink, effectively blocking the substrate‐binding domain and completely inhibiting DNAzyme activity. The crosslinks are then cleaved by 312 nm UV irradiation, allowing the substrate strand to hybridize to the DNAzyme via a strand displacement reaction. As a result, the activity of the DNAzyme can be precisely controlled by light.

Photoinactivatable DNAzymes have been constructed using blocker strands containing nucleobases protected with photolabile groups, namely, photocaged nucleobases. Deiters et al. synthesized a thymidine derivative bearing a 6‐nitropiperonyloxymethyl (NPOM) group at the N3 position of thymine [53], and incorporated it into the blocker strands (Figure 4f) [54]. The bulky protecting group prevented the blocker from hybridizing to the DNAzyme, thereby allowing the DNAzyme to retain its catalytic activity. UV irradiation removed the NPOM groups, allowing the blocker to hybridize to the substrate‐binding domain and part of the catalytic core, resulting in the inactivation of the DNAzyme upon light irradiation.

Reversible photoswitching of DNAzyme activity was also achieved by introducing photoisomerizable molecules (Figure 4g). Kamiya and Asanuma et al. rationally designed photoswitchable DNAzymes by blocking the catalytic loop with a complementary oligonucleotide containing photoresponsive azobenzene derivatives (Figure 4h) [55]. An RNA‐cleaving 10–23 DNAzyme [43] was conjugated to a blocker domain with azobenzene residues linked via D‐threoninol monomer unit [56]. When the azobenzenes are in the *trans* form, the blocker domain stably hybridized to the loop region, rendering the DNAzyme inactive. Photoisomerization to the sterically hindered *cis* form induced dissociation of the blocker domain and restored RNA‐cleaving activity, with the most successful case estimated to have an on–off ratio of 51 based on the initial reaction rate. A more efficient photoresponsive DNAzyme was developed by extending the blocker to bind not only to the catalytic loop but also to one of the substrate‐binding arms [55]. The blocking domain contains 2,6‐dimethyl‐4‐(methylthio)azobenzene residues, which undergo *cis–trans* isomerization under harmless visible light [57]. The on–off switching efficiency was improved to nearly 100‐fold, and the activity of the DNAzyme was reversibly controlled by alternating irradiation with two different wavelengths. This photoresponsive RNA‐cleaving DNAzyme was also applied to light‐dependent protein expression control [55].

## 5 Stimuli‐Responsive Caged DNAzymes

Caged nucleic acids are nucleic acids chemically modified with a protecting group (called a caging group) that can be removed by specific stimuli such as light, chemicals, or enzymes [58, 59, 60]. Caging groups temporarily inhibit the native function of DNA or RNA, and their removal (called uncaging) enables stimuli‐responsive activation. A variety of stimuli‐responsive caged DNAzymes have been designed by introducing caging groups into the substrate, substrate‐binding arms, or catalytic core.

**FIGURE 5:**
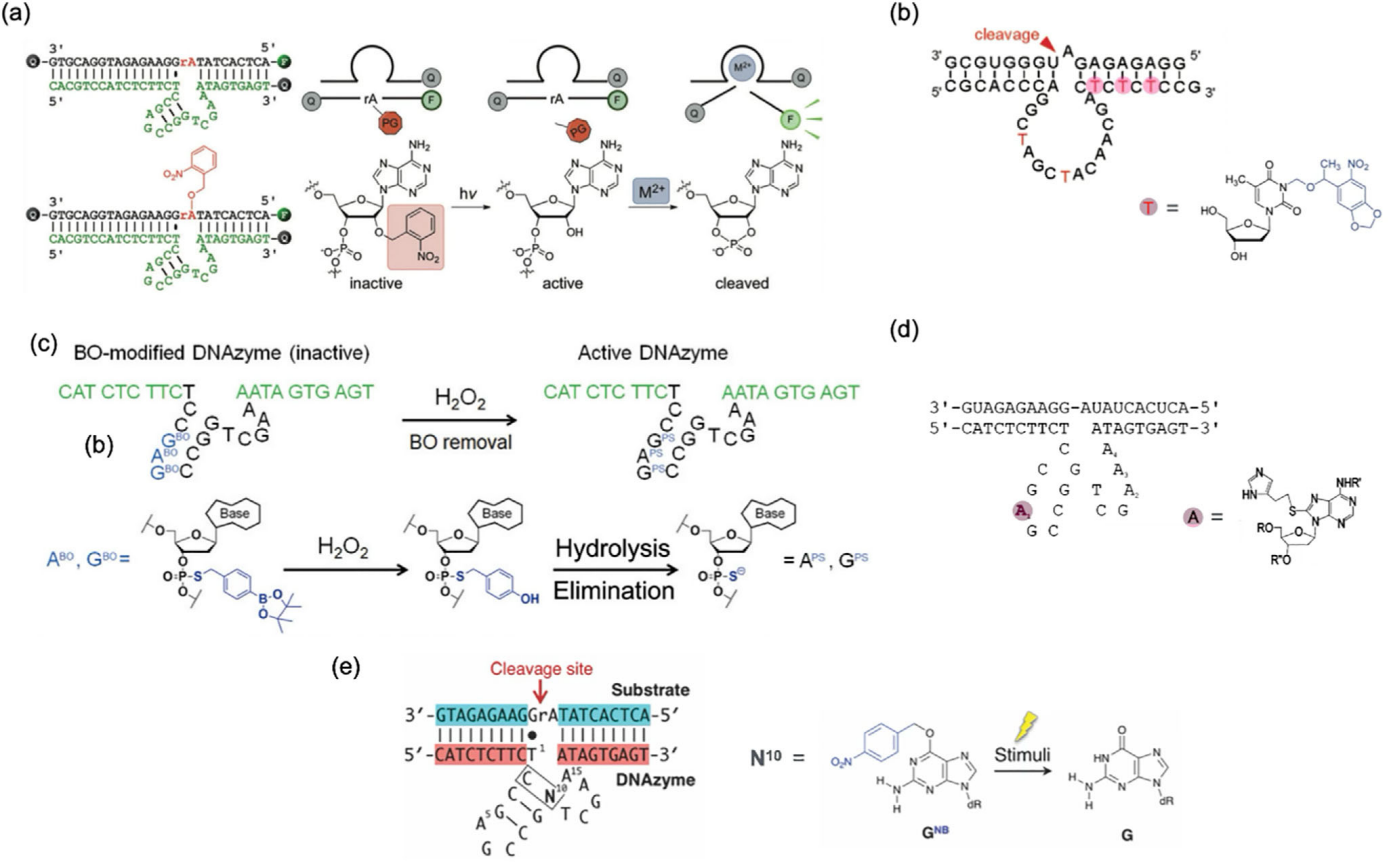
Molecular design of stimuli‐responsive caged DNAzymes. (a) A DNAzyme with a caged RNA substrate. The 2’‐OH group of the ribonucleotide (rA) at the cleavage site is protected with a photoremovable *o*‐nitrobenzyl group. F = fluorophore, Q = quencher, PG = photoremovable *o*‐nitrobenzyl group. Reproduced with permission from ref. [61]. Copyright 2014 Wiley‐VCH. (b) A photocaged DNAzyme containing an NPOM‐protected nucleobase at the catalytic core. Adapted with permission from ref. [53]. Copyright 2007 American Chemical Society. (c) An H_2_O_2_‐activatable DNAzyme whose catalytic core is modified with three phenylboronate (BO) groups. The mechanism of the H_2_O_2_‐induced uncaging is also shown. Reproduced with permission from ref. [48]. Copyright 2019 Wiley‐VCH. (d) A photoresponsive DNAzyme containing a photocaged adenosine analog in the catalytic core. Adapted with permission from ref. [66]. Copyright 2004 American Chemical Society. (e) A reducing agent‐responsive DNAzyme containing an *O* ^6^‐nitrobenzyl guanine (G^NB^) nucleobase. Reproduced with permission from ref. [68]. Copyright 2019 The Royal Society of Chemistry.

One effective strategy to control DNAzyme activity is to render the RNA substrate uncleavable by a caging strategy, which is achieved by protecting the 2’‐OH group of ribonucleotide at the scissile position, preventing nucleophilic attack on the phosphodiester linkage (Figure 5a) [39, 61]. Uncaging the substrate with a specific stimulus renders it cleavable, triggering the DNA‐catalyzed cleavage reaction. For example, the 2’‐OH group is protected with a photoresponsive *o*‐nitrobenzyl group, which can be removed by UV irradiation [62]. This strategy has been applied to the regulation of various RNA‐cleaving DNAzymes, including 8–17 [61], GR‐5 [61], and NaA43 DNAzymes [39], and hammerhead ribozymes [63]. Since the DNAzymes function only in the presence of metal ion cofactors, UV‐induced DNAzyme activation has enabled the spatiotemporal detection of metal species within cells [61, 64, 65].

Another simple approach to designing caged DNAzymes is to protect the substrate‐binding domain. Deiters et al. introduced a photoremovable 6‐nitropiperonyloxymethyl (NPOM) group at the N3 position of thymidine bases (Figure 5b) [53]. This chemical modification was predicted to disrupt the hydrogen‐bonded base pair with the RNA substrate and inhibit the DNAzyme reactions. When only one thymine base was caged, the activity of the DNAzyme was moderately reduced, but when three thymine bases were caged with NPOM, complete inhibition was observed. Brief UV irradiation at 365 nm uncaged the substrate‐binding arm and restored the activity of the DNAzyme.

Caging of the catalytic domain is also an approach to confer stimuli‐responsiveness to DNAzymes. Xing et al. developed a DNAzyme that responds to oxidizing species using phenylboronate (BO), which can be uncaged by H_2_O_2_ (Figure 5c) [48]. An 8–17 DNAzyme [43] was redesigned by introducing three BO‐protected phosphorothioate linkages into its catalytic core. The presence of inhibitory BO groups efficiently suppressed the activity of the DNAzyme, but treatment with 100 µM H_2_O_2_ increased the activity 41‐fold. It was also demonstrated that the BO‐protected DNAzyme was specifically activated by H_2_O_2_ even in the intracellular environment.

Perrin et al. developed a photolabile adenosine analog, 8‐(2‐(4‐imidazolyl)ethyl‐1‐thio)‐2’‐deoxyriboadenosine, which can be converted to native deoxyadenosine upon UV irradiation [66]. The modified adenine base was introduced into the catalytic loop of 8–17E DNAzyme (Figure 5d) [43]. Although the caged DNAzyme did not catalyze RNA cleavage, its catalytic activity was restored by UV irradiation. The aforementioned NPOM‐protected thymine base was also utilized in the development of a photoactivatable 10–23 DNAzyme [53]. Introducing a caged thymine into the catalytic domain in place of T12, which is known to be important for catalysis [67], completely inhibited the DNAzyme activity. UV irradiation to remove the caging group restored full DNAzyme activity. In contrast, caging a thymine at another position (T16) failed to inactivate the DNAzyme, highlighting the importance of selecting the appropriate position for caging.

Ikeda et al. developed a stimuli‐responsive DNAzyme using an *O* ^6^‐nitrobenzyl guanine (G^NB^) nucleobase (Figure 5e) [68], which can be uncaged by a reducing agent [69]. The G^NB^ nucleobase was introduced into the catalytic core of an RNA‐cleaving 8–17 DNAzyme [43]. Specifically, one of the guanine (G) base in the stem region was replaced with a G^NB^ to inactivate the DNAzyme. The addition of a reducing agent Na_2_S_2_O_4_ significantly enhanced the activity of the DNAzyme through reductive deprotection of the nitrobenzyl group. This result confirmed that the original catalytically active structure could be recovered by uncaging the G^NB^ base to form an intact G base.

**FIGURE 6:**
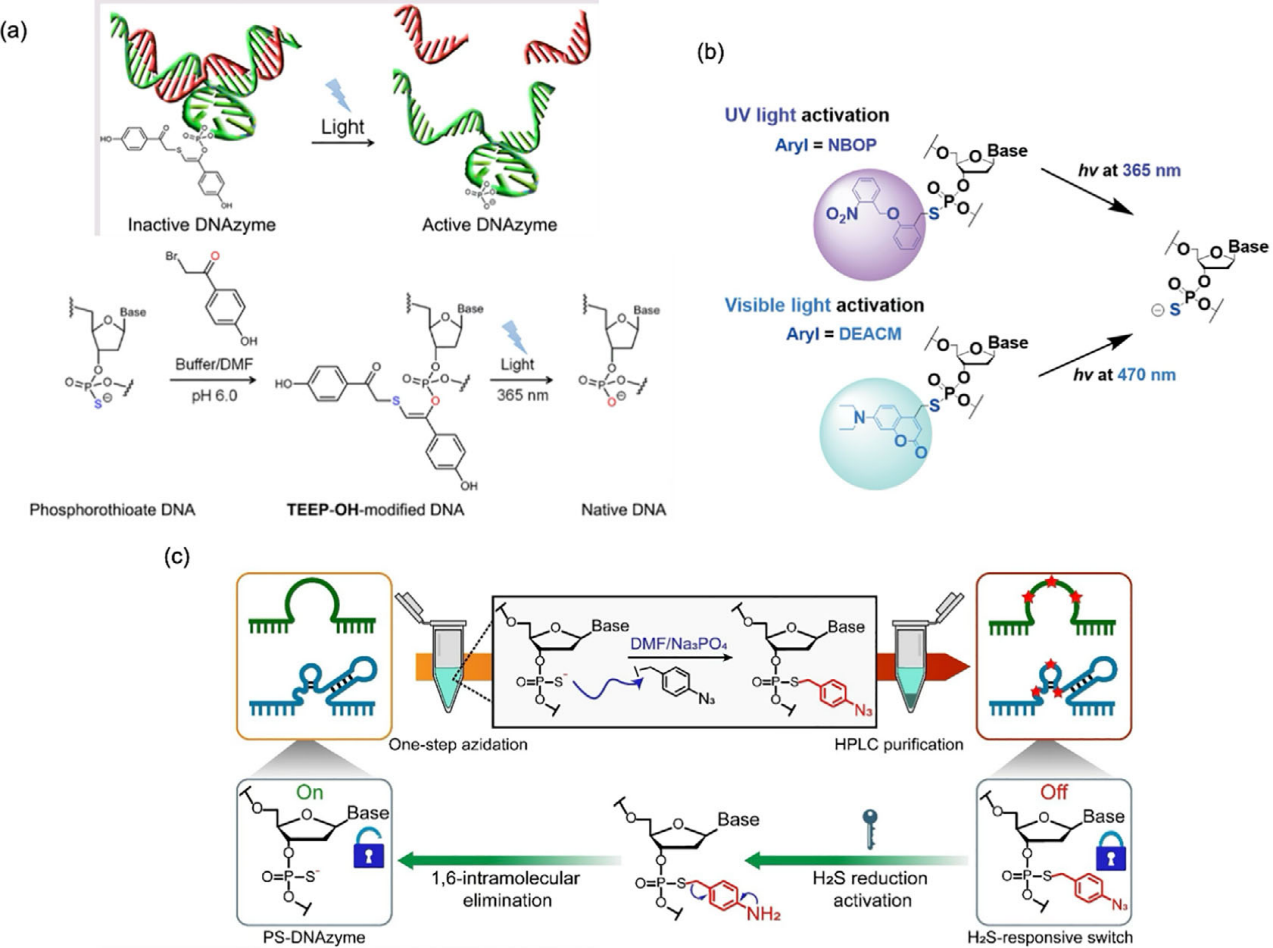
Molecular design of caged DNAzymes prepared via postsynthetic modification. (a) A photoactivatable DNAzyme constructed by postsynthetic introduction of a thioether‐enol phosphotriester (TEEP‐OH) group. Reproduced with permission from ref. [70]. Copyright 2016 American Chemical Society. (b) Activation mechanisms of 2‐(2‐nitrobenzyl)oxyphenyl (NBOP)‐ and 7‐diethylaminocoumarin (DEACM)‐modified DNAzymes upon UV irradiation at 365 nm and visible light irradiation at 470 nm, respectively. Reproduced with permission from ref. [71]. Copyright 2021 American Chemical Society. (c) Construction of H_2_S‐responsive DNAzymes through postsynthetic modification of phosphorothioates. H_2_S treatment reduces the azide groups, inducing removal of the caging groups. Reproduced with permission from ref. [73]. Copyright 2024 Wiley‐VCH.

Postsynthetic introduction of caging groups is a practical strategy for synthesizing stimuli‐responsive DNAzymes (Figure 6). Phosphorothioate groups, which can be easily incorporated into DNA backbones by standard solid‐phase synthesis, were used as reactive handles for site‐specific postsynthetic modification. Xiang et al. reported that reaction with 2‐bromo‐4’‐hydroxyacetophenone generates a photoremovable thioether‐enol phosphotriester group (TEEP‐OH) (Figure 6a) [70]. Three TEEP‐OH groups were introduced into the catalytic loops of the 8–17 and 10–23 DNAzymes [43], thereby suppressing their activity. Irradiation at 365 nm removed the caging groups, restoring the native phosphodiester linkages and reactivating the DNAzymes in a light‐dependent manner. Furthermore, caged DNAzymes were developed by postsynthetic introduction of a 2‐(2‐nitrobenzyl)oxyphenyl (NBOP) group or a 7‐diethylaminocoumarin (DEACM) group, which can be activated by UV or visible light irradiation, respectively (Figure 6b) [71]. The uncaged DNAzymes retained phosphorothioate linkages, yet exhibited significant recovery of activity. Furthermore, wavelength‐selective activation of these two DNAzymes was demonstrated in living cells.

Similarly, DNAzymes activatable by biorthogonal phosphine compounds and intracellular metabolites such as H_2_O_2_ and selenocysteine were strategically designed by postsynthetic introduction of corresponding caging groups [72, 73]. For example, biologically relevant hydrogen sulfide (H_2_S)‐responsive DNAzymes were developed by postsynthetic modification of the phosphorothioate backbone (Figure 6c) [73]. A 4‐azidobenzyl group was employed as the caging group, which can be removed by H_2_S‐mediated reduction. Three RNA‐cleaving DNAzymes gained H_2_S responsiveness by introducing three or four caging groups into their catalytic domain. These caged DNAzymes have been further applied in controlling cellular functions, such as inducing apoptosis and promoting proliferation.

**FIGURE 7:**
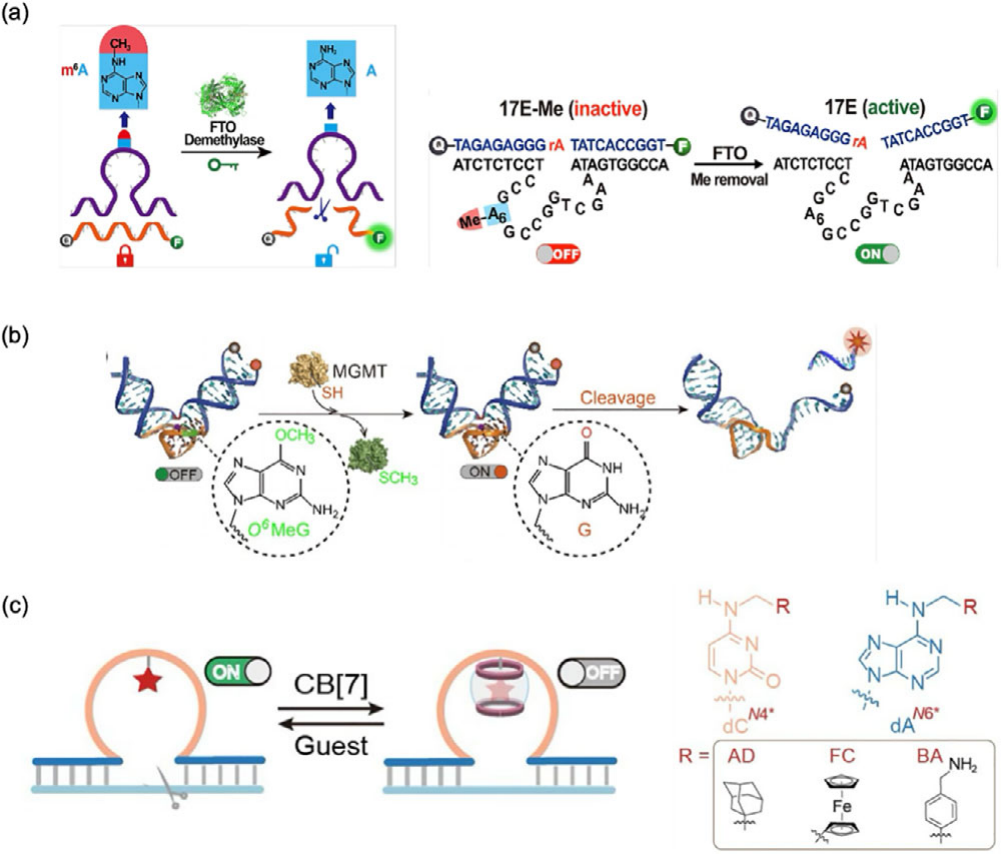
Schematic diagrams of stimuli‐responsive DNAzymes activated by enzymatic and host–guest chemistry reactions. (a) Enzymatic activation of DNAzymes via FTO (fat mass and obesity‐associated protein)‐mediated demethylation of *N* ^6^‐methyladenine (m^6^A) modifications. The base sequence of the m^6^A‐caged DNAzyme is also shown. Reproduced with permission from ref. [74]. Copyright 2021 American Chemical Society. (b) Enzymatic DNAzyme activation via MGMT (*O* ^6^‐methylguanine DNA methyltransferase)‐mediated demethylation of *O* ^6^‐methylguanine (O^6^MeG) modification. Reproduced with permission from ref. [77]. Copyright 2021 Wiley‐VCH. (c) Reversible control of DNAzyme activity through a host–guest recognition strategy. The catalytic core of the parent DNAzyme is modified with guest molecules (such as AD), to which the host molecule (cucurbit[7]uril (CB[7])) binds to inhibit the catalytic reaction. Addition of a competing guest molecule induces the dissociation of CB[7] from the DNAzyme, restoring its catalytic activity. Adapted with permission from ref. [78]. Copyright 2025 The Royal Society of Chemistry.

Naturally occurring methylated nucleobases have been utilized to create DNAzymes activated by specific enzymes, namely demethylases. Wang et al. introduced one of the epigenetically modified bases, *N* ^6^‐methyladenine (m^6^A), into the catalytic domain of the 8–17E DNAzyme (Figure 7a) [74]. The location of the m^6^A modification was determined by screening experiments, revealing that methylation of A6, located in the strictly conserved loop region [75], efficiently inactivated the DNAzyme. Because the methyl group of m^6^A can be removed by FTO (fat mass and obesity‐associated protein) [76], FTO‐mediated DNAzyme activation was demonstrated. Similarly, by introducing other methyl lesions, such as *O* ^6^‐methylguanine (*O* ^6^MeG), *N* ^1^‐methyladenine (1MeA), and *N* ^3^‐methylcytosine (3MeC), DNAzymes that respond to other types of demethylases were generated (Figure 7b) [77]. These caged DNAzymes have been applied to develop fluorogenic sensors for monitoring DNA repair activity in live cells, providing useful tools for biological research and clinical diagnostics. It is noteworthy that the abovementioned G^NB^‐modified DNAzyme was found to be activated by treatment with the DNA‐repair enzyme, *O* ^6^‐methylguanine DNA methyltransferase (MGMT) [68].

A new caging system based on host–guest interactions has recently been developed (Figure 7c) [78]. In this approach, guest molecules such as adamantane (AD) are introduced into natural nucleobases, such as the N4 position of cytosine or the N6 position of adenine. While these modifications only slightly affect base‐pairing properties, the binding of bulky host molecules (e.g., cucurbit[7]uril (CB[7]) [79]) interferes with base pairing, thereby disrupting and destabilizing the DNA duplex structures. To regulate the RNA‐cleaving activity of 10–23 DNAzyme [43], a guest‐modified nucleobase was incorporated into its catalytic domain. For example, replacing C10 with an AD‐modified cytosine did not significantly decrease the activity of the DNAzyme. However, upon addition of a CB[7] host molecule, the DNAzyme was efficiently inactivated, with the observed rate constant decreasing by more than 700‐fold. This result is attributed to the structural deformation induced by the binding of the large CB[7] molecule to the AD guest molecule. This “caged” DNAzyme was reactivated by adding a competitive guest molecule to displace CB[7] from the DNAzyme. Caging and uncaging based on supramolecular interactions promise to be a novel approach to the design of stimuli‐responsive DNA systems [80].

## 6 Stimuli‐Responsive DNAzymes With Intramolecular Structural Transformation

The catalytic activity of DNAzymes can be regulated by stimuli‐dependent structural transformation. This approach was first explored by incorporating a pair of photoresponsive azobenzene residues into the stem duplex of the 8–17 DNAzyme [81]. Under single‐turnover conditions, UV irradiation induced *trans*‐to‐*cis* isomerization of the azobenzene moieties, increasing its catalytic activity by approximately fivefold. Subsequently, photoresponsive 10–23 DNAzymes [43] were developed by introducing a nucleotide modified with an azobenzene unit at the 2’ position [82]. This modification was performed on the catalytic core, with the expectation that the active structure would change upon photoisomerization. The multiturnover catalytic activity of the modified DNAzymes was enhanced by up to sixfold by UV irradiation, and reversible activity switching was also demonstrated. Although the detailed mechanism remains unclear, the sterically folded structure of the catalytic domain is thought to be changed by photo‐induced isomerization of azobenzene.

**FIGURE 8:**
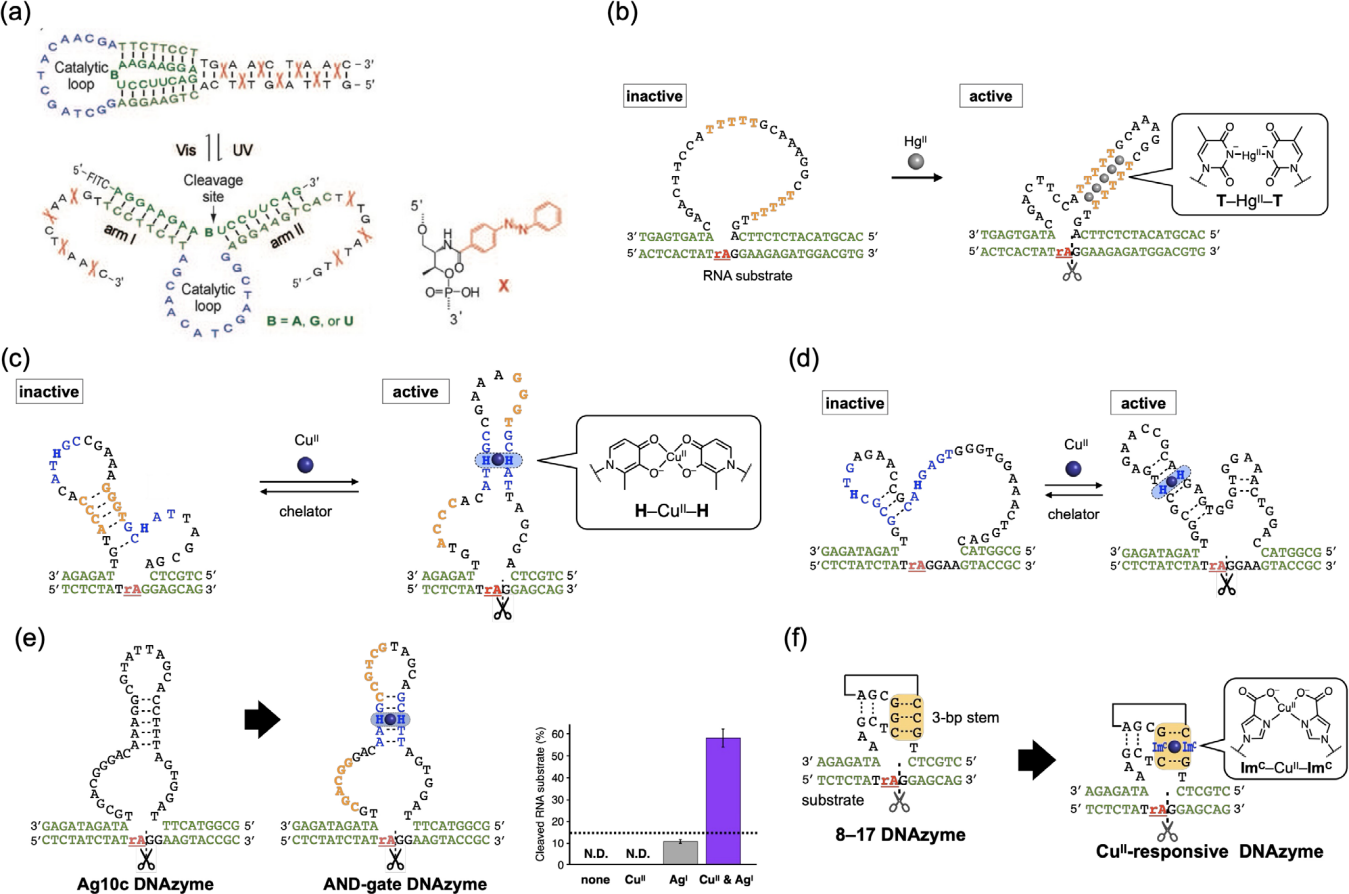
Molecular design of stimuli‐responsive DNAzymes that undergo intramolecular structural changes. (a) Photoresponsive DNAzyme containing azobenzene residues (indicated by X). Reproduced with permission from ref. 83. Copyright 2010 Wiley‐VCH. (b) Hg^II^‐responsive DNAzyme activated via T–Hg^II^–T base pair formation. “rA” in the substrate strand represents the adenine ribonucleotide at the cleavage site. (c) A Cu^II^‐responsive E5 DNAzyme activated via H–Cu^II^–H base pair formation. Both catalytically inactive and active secondary structures are shown. (d) A Cu^II^‐responsive Na43A DNAzyme activated via H–Cu^II^–H base pair formation. (e) Development of an AND‐gate DNAzyme responsive to Cu^II^ and Ag^I^ ions. The base sequences of the known Ag^I^‐dependent DNAzyme (Ag10c) and that of the AND‐gate DNAzyme containing an H–Cu^II^–H base pair are shown. (Right) RNA‐cleaving activity of the AND‐gate DNAzyme upon addition of Cu^II^ and/or Ag^I^ ions. [DNAzyme] = 1.0 µM, [substrate] = 10 µM, [CuSO_4_] = 0 or 1.0 µM, [AgNO_3_] = 0 or 10 µM in 10 mM HEPES buffer (pH 7.5), 200 mM NaNO_3_, 25 °C, 1 h, *N* = 3. Adapted with permission from ref. 88. Copyright 2020 American Chemical Society. (f) Development of a Cu^II^‐responsive 8–17 DNAzyme containing an Im^C^–Cu^II^–Im^C^ base pair. The base sequence of the original 8–18 DNAzyme is also shown. Hydrogen‐bonded base pairs (including the nonstandard A–G base pair) are indicated by broken lines. The 3‐bp short stem is highlighted. Adapted with permission from ref. 91. Copyright 2024 The Royal Society of Chemistry.

Asanuma et al. constructed a photoactivatable DNAzyme that undergoes more dynamic structural changes [83]. The photoresponsive DNAzyme was designed by connecting both ends of a 10–23 DNAzyme [43] with azobenzene‐containing sequences that are complementary to each other (Figure 8a). When the azobenzene residues adopt the *trans* configuration, these additional domains form a stable duplex, thereby distorting the overall DNAzyme structure. Upon UV irradiation, the azobenzene units are isomerized to the *cis* form, leading to dehybridization of the duplex, thereby restoring the catalytically active structure. The results demonstrated clear photoregulation of DNAzyme activity. Alternating irradiation with UV and visible light enabled reversible and repetitive regulation.

Metal‐responsive allosteric DNAzymes have been also developed based on intramolecular structural changes induced by metal‐mediated base pairing. Lu et al. constructed an Hg^II^‐responsive DNAzyme [84] by introducing Hg^II^‐mediated T–Hg^II^–T base pairs [85, 86] into the sequence of a UO_2_^2+^‐dependent RNA‐cleaving DNAzyme (Figure 8b) [87]. One to six T–T mismatches were inserted into the stem region of the parent DNAzyme, and the catalytically active structure was expected to form only upon the formation of T–Hg^II^–T base pairs. All modified DNAzymes exhibited higher RNA‐cleaving activity in the presence of Hg^II^ ions than in their absence. In particular, a DNAzyme with five T–T pairs showed a 153‐fold increase in activity upon the addition of Hg^II^. This system was further applied as a fluorescent catalytic beacon by labeling the substrate with a fluorophore and a quencher. Hg^II^‐induced DNAzyme activation enhanced fluorescence, enabling selective detection of Hg^II^ ions with a detection limit of 2.4 nM.

Takezawa, Shionoya, and coworkers designed a series of Cu^II^‐responsive DNAzymes by incorporating a pair of metal‐chelating hydroxypyridone (H) nucleotides [34] into the stem duplex of the parent DNAzymes (Figure 8c) [88]. In addition, the loop region was engineered to be complementary to a segment of the catalytic core. This design blocks the catalytic domain, thereby inactivating the DNAzyme in the absence of Cu^II^ ions. Upon H–Cu^II^–H base pairing, the structure undergoes a conformational change from an inactive to an active form, resulting in Cu^II^‐[dependent activation of the DNAzyme. For example, an allosteric DNAzyme designed based on the E5 DNAzyme [32] exhibited reduced RNA‐cleaving activity in the absence of Cu^II^ ions, but the addition of an equimolar amount of Cu^II^ enhanced its activity by 6.8‐fold [88]. This activation efficiency exceeds that of the H‐modified split DNAzyme described above [33]. Fluorescence assays using an H‐modified DNAzyme containing a fluorescent pyrrolocytosine residue demonstrated that the addition of Cu^II^ induced a structural change, verifying the intended mechanism of action. One equivalent of Cu^II^ was sufficient to activate the modified DNAzyme, and further addition of Cu^II^ did not increase activity. Furthermore, the activity of the DNAzyme could be reversibly regulated by the sequential addition and removal of Cu^II^ ions under isothermal conditions. Similarly, a modified E5 DNAzyme incorporating three consecutive H–Cu^II^–H base pairs was also developed, although it exhibited a modest response to Cu^II^ ions [89].

The same strategy has been used to confer metal‐responsiveness to other DNAzymes [88]. For example, the Na43A DNAzyme [39] was modified by introducing an H–H mismatch in the stem region (Figure 8d). In addition, the stem duplex was shortened so that the DNAzyme would adopt a catalytically inactive conformation in the absence of Cu^II^. The addition of one equivalent of Cu^II^ ions increased the activity of the modified DNAzyme by 5.9‐fold. This result indicates that the formation of an H–Cu^II^–H base pair realizes Cu^II^‐dependent allosteric regulation. Furthermore, the Ag^I^‐dependent RNA‐cleaving DNAzyme Ag10c [90] was engineered to respond to Cu^II^ ions by incorporating an H–H mismatch (Figure 8e) [88]. The engineered DNAzyme showed sufficient activity only in the presence of both Cu^II^ and Ag^I^ ions, demonstrating an AND logic gate response.

Recently, one of the smallest DNAzymes, the 8–17 DNAzyme [43], lacking a long stem duplex, was redesigned to function as a Cu^II^‐responsive DNAzyme (Figure 8f) [91]. The modification site was determined based on the reported crystal structure [92]. A pair of ligand‐type Im^C^ nucleotides [36, 37] was introduced into the center of the 3‐base‐pair duplex within the pseudoknot motif so that the formation of an Im^C^–Cu^II^–Im^C^ base pair induces folding into a catalytically active structure. Although structural modifications of highly compact nucleic acid motifs usually tend to inhibit their intrinsic activity, the modified DNAzyme showed a 5.1‐fold increase in activity upon addition of Cu^II^, demonstrating excellent metal responsiveness.

**FIGURE 9:**
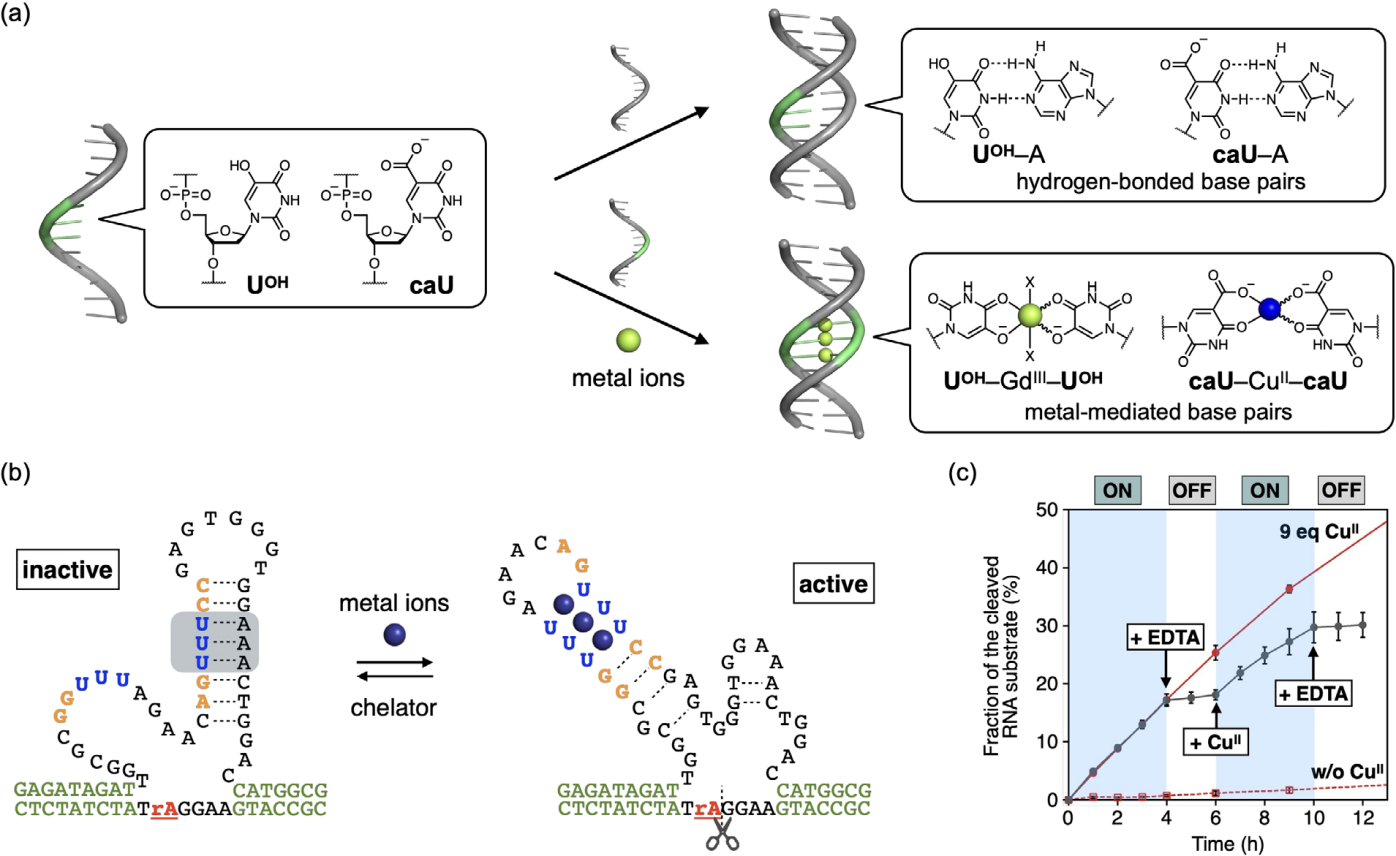
(a) Bifacial uracil bases modified at the 5‐position capable of forming both hydrogen‐bonded and metal‐mediated base pairs. The base pairing properties of 5‐hydroxyuracil (U^OH^) and 5‐carboxyuracil (caU) are shown. “X” represents additional coordinating ligands such as water molecules or adjacent nucleobases. (b) Molecular design of metal‐responsive DNAzymes using bifacial nucleobases. X = U^OH^ or caU. The active structures are formed through the formation of three U^OH^–Gd^III^–U^OH^ or caU–Cu^II^–caU base pairs, while the inactive structures contain three U^OH^–A or caU–A base pairs. “rA” in the substrate indicates an adenosine ribonucleotide at the cleavage site. (c) Iterative switching of the RNA‐cleaving activity of the caU‐modified DNAzyme. Cu^II^ (9 equiv relative to the DNAzyme) and EDTA (9 equiv) were added alternately. [DNAzyme] = 1.0 µM, [substrate] = 10 µM in 10 mM HEPES buffer (pH 7.0), 100 mM NaCl, 25 °C. DNAzyme activities in the absence (red dotted lines) and presence of Cu^II^ ions (red solid lines) are also shown. Reproduced with permission from ref. [99]. Copyright 2024 The Royal Society of Chemistry.

Takezawa and Shionoya proposed a new strategy for designing metal‐responsive allosteric DNAzymes by utilizing bifacial nucleobases that can form both hydrogen‐bonded and metal‐mediated base pairs [93, 94, 95, 96]. To this end, 5‐modified uracil bases, such as 5‐hydroxyuracil (U^OH^) [93, 94, 95] and 5‐carboxyuracil (caU) [96], were used (Figure 9a). As expected, these nucleobases form Watson–Crick‐like hydrogen‐bonded base pairs with natural adenine (A) base (e.g., U^OH^–A, caU–A). In contrast, these bases can also form metal‐mediated unnatural base pairs (e.g., U^OH^–Gd^III^–U^OH^, caU–Cu^II^–caU) in the presence of certain metal ions, because the carbonyl group at the 4‐position and the substituent at the 5‐position function as bidentate metal‐binding sites. This indicates that the base pairing partners of U^OH^ and caU bases can be switched by the addition or removal of the corresponding metal ions.

Taking advantage of this unique property, metal‐responsive DNAzymes were rationally designed (Figure 9b) [97]. Three consecutive U^OH^–U^OH^ mismatches were incorporated into the stem duplex of the NaA43 DNAzyme [39]. Furthermore, the surrounding base sequences were redesigned to block the catalytic core by U^OH^–A base pairing under Gd^III^‐free conditions. The modified DNAzyme was thus designed to undergo a structural change from an inactive form to an active conformation containing U^OH^–Gd^III^–U^OH^ base pairs. The base sequence was optimized using the secondary structure prediction software NUPACK [98], with the unnatural U^OH^–A and U^OH^–Gd^III^–U^OH^ base pairs virtually replaced by canonical T–A and G–C pairs, respectively. The RNA‐cleaving activity of the U^OH^‐modified DNAzyme increased 14.4‐fold with the addition of 3 equivalents of Gd^III^ ions (relative to the DNAzyme), whereas that of the unmodified DNAzyme increased only 1.9‐fold. UV spectroscopy confirmed the formation of U^OH^–Gd^III^–U^OH^ base pairs upon metal addition. It is also noteworthy that no activity was detected in a control DNAzyme in which all U^OH^ bases were replaced with thymine. These results suggest that the U^OH^‐modified DNAzyme is allosterically activated via the formation of U^OH^–Gd^III^–U^OH^ base pairs. Moreover, the activity of the DNAzyme could be reversibly regulated by the addition of Gd^III^ ions and the chelating agent EDTA.

Similarly, a Cu^II^‐responsive DNAzyme was developed based on switching between caU–A and caU–Cu^II^–caU base pairs (Figure 9b) [99]. The engineered DNAzyme was designed to adopt an inactive structure through caU–A base pairing and revert to its original active structure upon caU–Cu^II^–caU base pairing. As a result, the RNA‐cleaving activity of the caU‐modified DNAzyme increased 21‐fold upon the addition of Cu^II^ ions and was reversibly controlled by the addition and removal of Cu^II^ (Figure 9c). It is noteworthy that these metal‐responsive bifacial nucleobases have also been applied to metal‐triggered DNA strand displacement reactions and the construction of DNA tweezers that open and close in response to metal ions [97].

## 7 Summary and Outlook

This review outlines the rational design of stimuli‐responsive DNAzymes, focusing in particular on strategies that incorporate modified or artificial nucleotides to confer stimuli‐responsiveness to existing DNAzyme sequences. The most straightforward approach utilizes stimuli‐dependent control of DNA hybridization, enabling the reassembly of split DNAzyme fragments or the temporary blocking and unblocking of their catalytic core and substrate‐binding arms. Another related strategy is the control of DNAzyme activity through strand cleavage and ligation. This can be considered a variation of the split DNAzyme approach, in which functional reconstitution is controlled by the formation or cleavage of chemical bonds within the DNA backbone. Because various types of stimuli‐responsive units can be introduced, these modular design strategies provide a versatile framework for constructing DNAzymes that respond to a wide range of external stimuli and environmental changes.

Another important design approach is the use of caged nucleic acids [58, 59, 60], which utilizes protecting groups that can be removed by external stimuli to control DNAzyme activity. Although the uncaging process is generally not reversible, this approach allows for direct suppression of the catalytic site or inhibition of substrate binding, making it widely applicable to various DNAzyme systems.

On the other hand, strategies based on reversible changes in intramolecular DNA folding are more sophisticated and challenging approaches. These systems are designed to stably adopt catalytically active or inactive conformations in response to external stimuli. This concept is similar to the regulatory mechanism of allosteric protein enzymes, where structural changes induced by effector binding modulate catalytic activity. Such designs provide the possibility of higher‐order control, going beyond simple on/off control, including stimuli‐dependent switching of substrate specificity and other complex functional behaviors.

The development of stimuli‐responsive DNAzymes has utilized a variety of functional molecules, including caged nucleobases, backbone modifications, and side chains with intercalating properties. These components undergo structural changes or deprotection reactions in response to light, chemicals, and other external stimuli, allowing precise control of DNAzyme activity from the outside. Recent advances have further demonstrated the utility of novel design elements, such as host–guest chemistry‐based systems [78], that enable reversible control through noncovalent interactions. Furthermore, artificial base pairs that selectively form in the presence of specific chemical species or environmental stimuli (e.g., metal‐mediated base pairs [24, 25]) have emerged as valuable tools for the rational design of responsive nucleic acid systems. These strategies utilize chemical modifications to existing DNAzyme sequences, providing a rational and modular framework for functional design. Notably, such approaches are not limited to DNAzymes, but can be broadly applied to other functional nucleic acids, as well as to DNA‐based supramolecular systems and nanostructures [100, 101], enabling stimuli‐triggered structural and functional transformations.

DNAzymes, especially those capable of RNA cleavage, have attracted increasing attention as programmable catalysts for a wide range of applications, from biosensing and diagnostics to molecular computing and nanotechnology [10, 11, 12, 13, 14]. Compared with conventional DNAzymes, stimuli‐responsive DNAzymes offer distinct advantages by enabling conditional control of catalytic activity in response to specific chemical, biological, or physical inputs. In principle, the rational design strategies for stimuli‐responsive DNAzymes discussed in this review can further enhance their controllability and programmability. For example, such responsiveness enables precise spatiotemporal regulation of RNA cleavage, thereby minimizing uncontrolled background activity. This feature is particularly valuable for applications in complex environments, including biological systems. As discussed above, stimuli‐responsive DNAzymes provide highly selective sensing platforms. In particular, they can be integrated with DNA logic gates, signal amplification systems, and DNA nanodevices, making them powerful tools for advanced biosensing and diagnostic assays. Moreover, externally triggered activation with fine temporal resolution will greatly contribute to the development of DNA molecular machines and advances in dynamic DNA nanotechnology.

Despite these advantages, several challenges remain. Many stimuli‐responsive DNAzymes still suffer from incomplete on/off switching, inefficient response kinetics, and limited robustness under physiological conditions. In addition, precise control over the effective concentration ranges of triggering molecules or metal ions remains a critical issue for practical applications. Designing DNAzymes that respond predictably to multiple inputs is still challenging, although such multiinput responsiveness is essential for logic gate‐based molecular computing as well as smart therapeutic systems. Consequently, further exploration of rational design strategies will become increasingly important for improving both the performance and applicability of stimuli‐responsive DNAzymes. For biological applications, achieving adequate in vivo stability and efficient delivery is also a key issue to be overcome, as is the case for other functional nucleic acids. Furthermore, the integration of stimuli‐responsive DNAzymes into higher‐order systems, such as DNA computational circuits and DNA nanodevices, represents a crucial next step toward the construction of more complex and functional molecular systems. Addressing these challenges will require not only improved strategies for base‐sequence design but also the effective exploitation of chemically modified and artificial nucleotides.

The continued development of new chemical modifications and response motifs will enable DNAzymes to respond to a wider range of stimuli with greater selectivity and sensitivity. Furthermore, combining stimuli‐responsive DNAzymes with DNA nanotechnology and synthetic biological systems may allow the creation of more sophisticated, adaptive, and autonomous molecular functions. Ultimately, the integration of such DNAzymes into complex molecular networks could drive future advances in molecular computing and molecular robotics [102, 103]. Accordingly, continued progress in the rational design of stimuli‐responsive DNAzymes will open up new frontiers in all areas of DNA nanotechnology, therapeutics, and synthetic biology.

## Conflicts of Interest

The authors declare no conflict of interest.
